# Teammates Instead of Tools: The Impacts of Level of Autonomy on Mission Performance and Human–Agent Teaming Dynamics in Multi-Agent Distributed Teams

**DOI:** 10.3389/frobt.2022.782134

**Published:** 2022-05-20

**Authors:** Summer Rebensky, Kendall Carmody, Cherrise Ficke, Meredith Carroll, Winston Bennett

**Affiliations:** ^1^ Aptima, Fairborn, OH, United States; ^2^ College of Aeronautics, Florida Tech, Melbourne, FL, United States; ^3^ Air Force Research Laboratory, Dayton, OH, United States

**Keywords:** human agent teaming, level of autonomy, multi-agent teaming, distributed teams, autonomous decision making

## Abstract

Human–agent teaming (HAT) is becoming more commonplace across industry, military, and consumer settings. Agents are becoming more advanced, more integrated, and more responsible for tasks previously assigned to humans. In addition, the dyadic human–agent teaming nature is evolving from a one–one pair to one–many, in which the human is working with numerous agents to accomplish a task. As capabilities become more advanced and humanlike, the best method for humans and agents to effectively coordinate is still unknown. Therefore, current research must start diverting focus from how many agents can a human manage to how can agents and humans work together effectively. Levels of autonomy (LOAs), or varying levels of responsibility given to the agents, implemented specifically in the decision-making process could potentially address some of the issues related to workload, stress, performance, and trust. This study sought to explore the effects of different LOAs on human–machine team coordination, performance, trust, and decision making in hand with assessments of operator workload and stress in a simulated multi-unmanned aircraft vehicle (UAV) intelligence surveillance and reconnaissance (ISR) task. The results of the study can be used to identify human factor roadblocks to effective HAT and provide guidance for future designs of HAT. Additionally, the unique impacts of LOA and autonomous decision making by agents on trust are explored.

## Introduction

The exponential growth and benefits of automated systems have generated a lucrative and growing market for unmanned aerial systems (UASs), projected to reach $82.1 billion by 2025 (Barnhart et al., 2016). The major advantages for UASs include their compact size and quick deployability. UASs can support data gathering, transport, package delivery, medical supply delivery, emergency services, and military missions. UAS capabilities are also expanding to perform many warfighter tasks, including tactical intelligence surveillance and reconnaissance (ISR), supply delivery, cyber defense, and suppression of enemy air defenses (SEAD), and the future is likely to include UAS and humans working within multi-agent systems (U.S. Air Force, 2016; Chen & Barnes, 2014). The majority of UAS missions are dedicated to surveillance and reconnaissance uses (Schmitt & Stuetz, 2019). During ISR missions, humans must operate under rapid response times and simultaneously provide mission efficiency across the board (Wang et al., 2017). In multi-unmanned aircraft vehicle (multi-UAV) operations, the human controls or monitors a fleet of UAVs, allowing for more rapid and dynamic missions. This technological shift provides a promising outlook for human–agent teams (HATs), which sets a new path for autonomous systems, or agents, to be viewed as team members, rather than tools. By identifying methods to enhance human–agent cooperation through design, multi-UAV teams can be utilized to greatly increase mission performance in military operations.

Although there has been substantive research on a dyadic HAT, research in multi-HAT settings has focused on automating lower-level functions such as navigation and health monitoring (Ruff et al., 2002; Porat et al., 2016). Research with basic UAV automation has shown that as the number of UAVs increases, the level of autonomous decision making can impact human operator trust and how operators utilize the UAVs in their own decision making (Ruff et al., 2002; Nam et al., 2018). Utilization of two vehicles in a multi-HAT target-tracking task has been shown to lead to increased performance; however, studies have shown utilizing three or more vehicles becomes difficult for operators to manage and process (Porat et al., 2016; Humann & Pollard, 2019). Multi-UAV systems face many issues, including 1) overlapping and diversity of tasks, 2) allocation of tasks, 3) inconsistent and complex communication, 4) dynamic events such as an unpredictable environment, 5) continuous system monitoring, 6) issues with task switching and interruptions, 7) lack of information prioritization, 8) lack of displays that are centered around the task, and 9) lack of decision-aiding technology (Zhang & Xing, 2020; Fu et al., 2018; Da Silva et al., 2017). Furthermore, current research must start exploring the dynamics of HAT from a human factor and multi-agent perspective, as they perform more advanced capabilities such as tactical decision making and adaptive behaviors (Habib et al., 2017). More advanced agents require different methods and techniques to facilitate coordination between the system and human operators. Focusing on finding the upper limits of structure and team size for human operators to handle has led to a neglect of research focusing on improving the way the team works together. There are many considerations when pairing multiple UAV agents with humans as teammates with respect to directability, transparency, collaboration, predictability, availability of status and intentions, and adaptability to new conditions (Christoffersen and Woods, 2002; Klein et al., 2004; Humann and Pollard, 2019). Therefore, current research must start diverting focus from “how many systems can a single operator control?” to “how to distribute missions among operators and systems in an efficient manner?” (Porat et al., 2016, p.1).

Increasing the tasking load to the agent, otherwise known as level of autonomy (LOA), by offloading tasks such as navigation and object recognition, could result in reductions in operator workload while also increasing the maximum number of UAVs an operator could manage without performance decrements (Humann and Pollard, 2019). LOA is defined as the range of design options implemented in a system to enhance self-sufficiency and self-directedness, ranging from manual operations which require humans to complete all functions, to fully autonomous operations, in which the system is able to perform the task in its entirety, requiring no assistance (Johnson et al., 2011; see Table 1). The trend in research for single-HAT suggests the key to running a smooth operation is to identify the appropriate calibration of task management amongst team members whilst providing operators with flexible automation tools to direct mission tasks (Eggers and Draper, 2006). However, as agents become increasingly intelligent, the number of agents a human can control increases and requires new considerations (Chen and Barnes, 2014). As HAT capabilities become more advanced and humanlike, the best method for humans and agents to effectively coordinate is still an open question and requires careful and methodological design of each LOA (Habib et al., 2017).

**TABLE 1:** Sheridan and Verplank’s (1978) LOA structured model.

Level of automation	Definition
1	Automation offers no assistance, humans must do it all
2	The computer offers a complete set of action alternative and nice
3	Narrows the selection down to a few, or
4	Suggests one, and
5	Executes that suggestion if the human approves, or
6	Allows the human a restricted time to veto before automatic execution, or
7	Executes automatically, then necessarily informs the human, or
8	Informs the human after execution only if asked, or
9	Informs the human after execution if the automation decides to
10	The automation decides everything and acts autonomously, ignoring the human

### Human–Agent Team Effectiveness Issues

The effectiveness of HAT is influenced by various factors including system transparency, communication methods, human trust in the agent, human workload level, situation awareness, and individual differences such as the ability to multitask (Strybel et al., 2018).

***Workload and performance.*** In the case of a single operator overseeing multiple agents in multi-HAT missions, cognitive overload may occur. During simulated ISR missions in which an operator controlled four vehicles which were navigating and communicating in a benign setting, workload was found to exceed the acceptable limit for a majority of the mission, resulting in mission degradation, particularly any time communication was needed (Schneider et al., 2011). Furthermore, workload can increase as the reliability of an autonomous teammate decreases as operators take more manual control (Chen and Barnes, 2012). Furthermore, other research has shown operators may become neglectful of monitoring agents during peaks of workload unrelated to the agent (Ruff et al., 2002).

As the autonomy of the agent increases, one would expect that workload would decrease; however, the nature of the operator’s tasks shifts to a more mental task as opposed to physical. Many studies have demonstrated that as the number of automated systems that require monitoring increases, negative impacts occur on various factors such as situation awareness, workload, and target detection—which can ultimately hinder performance (Salcedo et al., 2011; Chen and Barnes, 2012; Barnes et al., 2015). Other negative outcomes could include overreliance on automation, failure to maintain attention on automation, or complacency (Spravka et al., 2003). More research on multi-UAV teaming with more advanced autonomous capabilities and independent agents and the resulting impacts on performance and workload is needed. Based on the research in this area from a range of domains, we anticipated that higher levels of decision-making capabilities from the agent would result in reduced workload on the operator. However, the impact on mission performance may not be as clear.

***Trust.*** Trust in HAT can be defined as “the attitude that an agent will help achieve an individual’s goals in a situation characterized by uncertainty and vulnerability” (Lee and See, 2004, p. 54). Trust is a crucial factor that aids in increasing the efficiency between humans and autonomous agents, and without it, the performance of the team can suffer (Joe et al., 2014). Research has shown that achieving higher trust in HAT can be acquired through humanizing the way automation interacts with its teammates (Quinn et al., 2017).

Trust in an autonomous agent is related to the system elements such as reliability, individual factors such as willingness to trust, and situational factors such as task workload (Endsley, 2015; Guglielmelli, 2015). Relative to multi-HAT operations, the main drawbacks are the lack of transparency (also referred to as observability) and directability of the agent, which prevent UAVs from being perceived as teammates (Christoffersen and Woods, 2002). These deficiencies break down communication, and consequently, trust may decrease due to the lack of awareness relative to agent actions (Klein et al., 2004; Alaieri and Vellino, 2016). This can lead to a domino effect by increasing workload and stress, which may result in low mission performance (Joe et al., 2014). Reliability of an agent impacts HAT dynamics including decreased trust, higher stress, and poor mission performance with low reliability and a higher likelihood to comply with false alarms when high (Chen and Barnes, 2014; Schaefer et al., 2017; Wohleber et al., 2019). In the current study, depending on the LOA, characteristics such as transparency, reliability, and task allocation may impact the human operator in various ways such as overreliance or disuse. Therefore, it is important to investigate changes in the perceptions of the characteristics and related levels of trust to further understand how trust impacts mission performance, effectiveness, and team capabilities in different LOAs.

Chen and Barnes (2014) presented the concept of mixed-initiative autonomous systems which takes a more team-like decision making approach to HAT. Decisions are facilitated through interfaces to mimic the more collaborative environment of a human–human team (HHT). The agents can communicate their intents, which are reviewed and approved by a human operator. Mixed initiative requires team members to jointly make a decision regarding the developments in the tasking environment and supports bi-directional communication between the operator and the agents, which can help improve situation awareness, transparency, directability, and, ultimately, trust calibration (Chen and Barnes 2014; Schaefer et al., 2019). Research suggests that human operators require less effort, complete more tasks, improve trust, and improve mission performance when supported by mixed-initiative planning (Ramchurn et al., 2015; Nam et al., 2018). This team dynamic has shown itself to be superior specifically for tasks such as object detection—making it a desirable task to explore LOAs and mixed initiative design for ISR missions (Barnes et al., 2015). Therefore, the current study utilized a mixed-initiative HAT approach.

### Levels of Autonomy Impacts in Human–Agent Teams

LOAs implemented specifically in the decision-making process could potentially address some of the issues related to workload, stress, performance, and trust. Schneider et al. (2011) outlined how identifying the best LOA can aid in decreasing operator workload and stress in dynamically changing environments and, in return, increase mission effectiveness. Research by Ruff et al. (2002) found that performance gains are highest with middle-level LOAs in which the agent asks for consent to carry out actions as opposed to manual or fully autonomous agents. This LOA was most effective likely due to the lower LOA condition offering no assistance to relieve operator workload, and the high LOA condition not sufficiently keeping the human in the loop. Increasing automation to the extent that the human simply overrides potential actions removes the human more from the decision-making process, lowering SA and making it more difficult for the human to make decisions when it is necessary. Therefore, too much automation can allow the human to “slack off,” resulting in operators who are unaware of what is going on until it is too late (Ruff et al., 2002). Additional downsides to too much automation have been shown in studies in which high levels of workload in dynamically changing environments led to increased reliance on automation (Biros et al., 2004). Biros et al. (2004) discovered that even though automation trust and use have a positive relationship, increased workload can have a negative effect on this relationship, ultimately leading to use of the automation even when trust is low.

In a multi-UAV setting, this could lead to poor decision making based on incorrect UAV information, incidents, or accidents. This notion emphasizes that automation should be allocated based on the type of mission and the skill of the operator who is carrying out the mission (Eggers and Draper, 2006). Furthermore, Parasuraman et al. (2000) suggested that high LOAs could be implemented for information acquisition and information analysis. However, when considering high-risk functions and outcomes, the LOAs should be lower for actual decision making and action implementation as the outcomes could be more devastating if left to automation. Some researchers note that even if one chooses to follow guidance for a particular LOA, no guidance is provided on *how* to implement each LOA in an interface (Johnson et al., 2011). These findings suggest that the relationship between performance and LOA is more nuanced and cannot be simplified to a “more is better” scenario. As research suggests, implementing meaningful human control through HAT will allow human operators to fully benefit from a system’s autonomous capabilities (van Diggelen et al., 2019).

### Current Study

Determining the best LOA for HAT missions may lead to improved mission performance, overall mission effectiveness, and ease of communication in HATs. Agents are becoming increasingly autonomous, ranging from navigation, industry, and health, to decision making (Amanatiadis et al., 2015; Khatib, 2019; Zacharaki et al., 2021). Furthermore, more advanced agents in a multi-HAT may require new or different LOAs and communication methods between human and agent. As multi-UAV missions are becoming increasingly popular, it is important for researchers to identify the unique challenges multi-HAT faces compared to single-HAT. When operating under different LOAs, the makeup of the autonomy and approach to communicate status to the operator drastically change, requiring new forms of coordination, interdependence, and communication design (Johnson et al., 2011). As LOAs fluctuate throughout a mission, designers typically overlook how humans may adapt to changes in different LOAs and how to implement higher LOAs effectively (Johnson et al., 2011). This study sought to explore the impacts on a variety of mission-critical factors as a result of changing LOA.

According to Chen and Barnes (2014), the general nature of military operations leads to high-stress and high-workload environments that are prone to mistakes and errors. The responsibility for these mistakes will likely rest on the human operator. The human in a HAT will perform as a supervisor to agents, which can let the human operator focus on more complex tactical decisions, and the agents can perform the tasks more appropriate for agent technology. These most closely align with Sheridan and Verplank’s (1978) LOAs 1 (computer offers no assistance) through 7 (system executes and then informs the human), allowing agents to perform tasks and the human operator to act in a supervisory role. In a military setting, mixed-initiative systems that provide information to the operator and leave ultimate decision making up to the human could create a “synergy between humans and intelligent agents—but not an equivalency” (Chen and Barnes, 2014, p. 18). Level 5 (consent) was also utilized previously for target detection in a multi-UAV simulation task (Wohleber et al., 2019). Based on the expectation that UAV agents will serve as teammates for a human operator and will always be—in some form—supervised by the human for the foreseeable future, the current study did not include the LOA in which the agent acts without the human in the loop (levels 8 and above; Sheridan, 1992; Arciszewski et al., 2009; Chen and Barnes, 2014). Utilizing LOA frameworks from the studies by Sheridan and Verplank (1978) and Arciszewski et al. (2009), as well as Chen and Barnes’ (2014) mixed initiative concept, the current study evaluated a range of levels from one to seven to determine the impacts on mission performance, trust, and team effectiveness in a HAT mission. The current study sought to answer three research question areas:

1. How does LOA in UAV teammates impact human–teammate mission performance and operator state (target identification performance, stress, workload, and decision making) in an ISR scenario with multiple autonomous vehicles?
2. How does LOA in UAV teammates impact human–teammate trust in an ISR scenario with multiple autonomous vehicles?
3. How does LOA in UAV teammates impact human–teammate team effectiveness (perceived effectiveness and perceived coordination) in an ISR scenario with multiple autonomous vehicles?

## Methods

A total of 49 participants completed the study and were within the target age range of 18–40 years old. Eight participants were removed from the data set for various reasons. Of these, two participants were excluded due to low English proficiency, which could potentially impact their responses. Additionally, six participants were removed as outliers based on average trust (subjective) and distrust (manual pictures taken) scores being at least two standard deviations from the mean. The resulting 41 participants were utilized in the dataset. The overall average age was 24 years old, consisting of 24 males and 17 females. A total of 61% of participants were Caucasian, followed by 9.8% African American, 9.8% Asian, and 19.5% other. Participants’ UAS experience was distributed with 53.7% having no experience, 29.3% having less than 5 h of UAS experience, and 17% with more than 5 h of UAS experience. Participants’ video game experience was distributed with 26.8% having less than 6 months, 17.1% ranging between 1 year to 3 years, and 56.1% over 3 years of video game experience. 17.2% of participants stated that they play video games daily, followed by 26.8% weekly, 26.8% monthly, 7.3% yearly, and 21.9% who stated that they never play video games.

### Experimental Design

**FIGURE 1:**
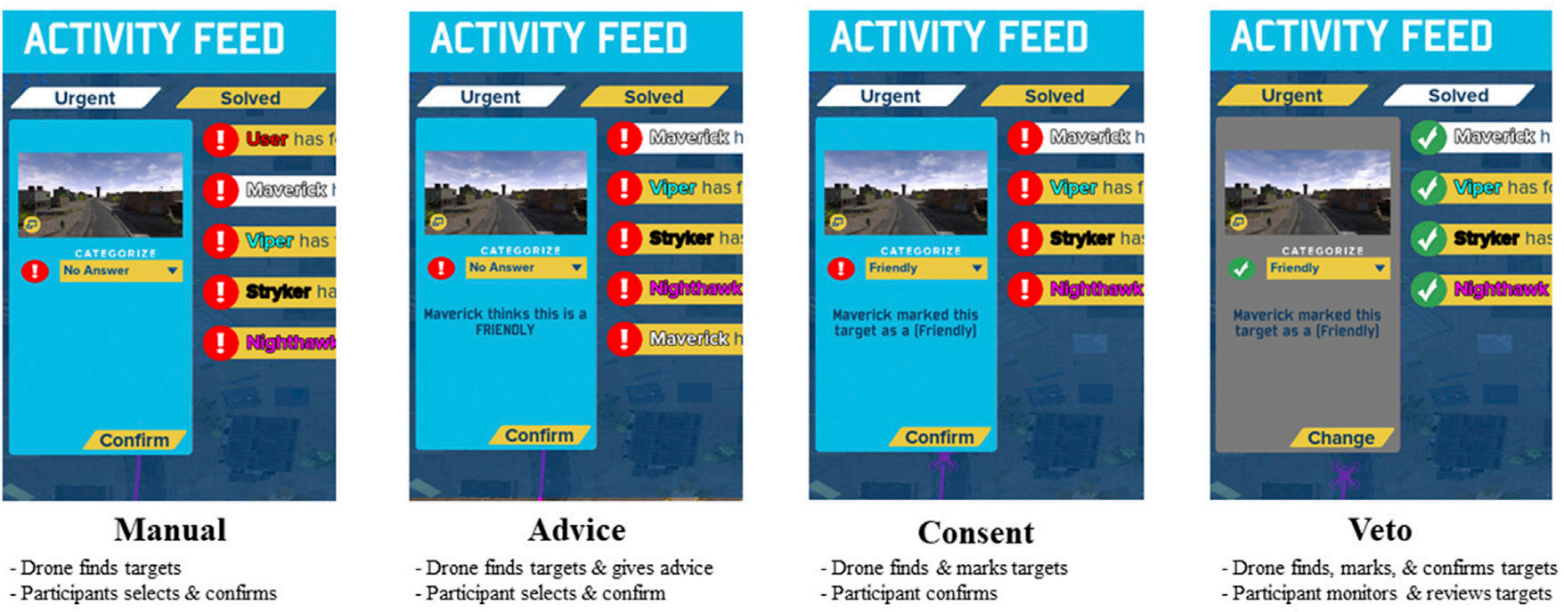
Experimental conditions.

The study design was a repeated measures within-subjects design. The independent variable was LOAs, which was manipulated using four levels (manual, advice, consent, and veto) presented in Table 2 and illustrated in Figure 1. The participants received all four autonomy levels (manual, advice, consent, and veto) in counterbalanced rotating orders. Seven dependent variables were evaluated, including team coordination, mission performance, team effectiveness, trust, stress, workload, and decision making. The experimental task included the participant monitoring four agent teammates whilst ensuring correct target detection across the mission.

**TABLE 2:** Level of autonomy conditions.

Level of autonomy	Level from Sheridan & Verplank (1978)	Agent responsibilities	Human involvement
Manual	1	Detects objects but does not offer any assistance with identification	Determines if the object is a friendly target, neutral target, or enemy target to update the mission map
	Computer offers no assistance		
Advice	4	Detects objects and offers suggestion on potential target type	Reviews agent suggestion and determines if the object is a friendly target, neutral target, or enemy target to update the mission map
	Suggests one		
Consent	5	Detects objects and marks target type	Reviews agent mark and either confirms or changes the agent’s decision
	Execute automatically if human approves		
Veto	7	Detects objects and marks target type	Can review the agent’s decision and change if needed
	Executes and then informs human		

The experiment asked participants to complete a series of four intelligence surveillance and reconnaissance missions with agents of four levels of autonomy. Participants worked with four agent teammates to take pictures of soldiers as targets and mark them as friendly, enemies, or not targets. When a picture was taken (either by the participant or the agent), the participant would need to decide on what type of target it was. The participant received varying levels of assistance with this decision-making task based on the level of autonomy condition. After each mission, participants filled out a series of surveys while performance information was collected automatically by the simulator.

In the manual condition, the agent only assisted with target detection. The agent would take pictures when enemy, friendly, or neutral assets were detected. After the agent found targets, a notification was placed in the urgent tab of the event manager in which the participant would select the target event, classify the target type as enemy, friendly, or neutral, and click confirm. Once the participant confirmed the target, the target event moved to the solved tab. In this condition, human involvement included classifying, selecting the classification category, and confirming every target.

In the advice condition, the agent detected the target, the same as in the manual condition. However, once a participant selected the target event, a message suggesting the target classification type was presented. For example, messages such as “Maverick thinks this is a FRIENDLY” or “Stryker thinks this is an ENEMY” were displayed. The participant then had to select the appropriate classification type and confirm the selection. Once the participant confirmed each target, the notification was moved into the solved tab. Human involvement included classifying, selecting the classification category (with advice from the agent), and confirming the target type. These were the same responsibilities as those included in the manual condition; however, participants had the option to follow the agents’ suggestions or make their own judgement calls.

In the consent condition, the agent detected the targets, the same as in the manual and advice conditions. However, once a participant selected the target event, the target classification type was already selected. For example, messages such as “Maverick marked this target as an ENEMY” or “Stryker marked this target as a FRIENDLY” were displayed with the target type preselected in the dropdown. Once the participant confirmed each target, the notification was moved into the solved tab. Human involvement included confirming or changing and confirming the classification category in the event manager. These were the same responsibilities as those included in the manual and advice conditions; however, participants only had to confirm the agent’s pre-selected target type or make their own judgement calls.

In the veto condition, the agent found, marked, and confirmed events. This automatically moved all target events into the solved tab. If a participant selected a target event in the solved tab, the same messages were presented as in the consent condition, but no action was required of the participant other than reviewing events if they chose to do so. If the participant felt the need to review and correct the agent’s decision, they were able to do so in the solved tab. Participants would select the new target type from the dropdown and click the change button. Human involvement included reviewing or changing the classification of the solved target events. This shifted the responsibilities to where the human was only responsible for double-checking agent target classifications if they chose to do so.

In all conditions, once a participant clicked on any events in the urgent or solved tab, a “reviewed” indicator would appear to inform the participant of which events they had already visited and which they had not. In addition to monitoring the event manager and camera feeds of the four agent teammates, the participants could also perform the target identification task manually in parallel as a backup to the agents if they believed the agent had missed a target. This would include manually taking pictures which would then be displayed on the urgent tab as “User has found something.” The manual picture events did not offer any agent assistance and were moved to the solved tab once the participant selected the target type and confirmed.

### Experimental Task

The experimental task was a military ISR mission in which the HAT was searching for enemies along a series of routes to identify the safest route to send a convoy. Participants were exposed to two separate screens. The left screen displayed a mission map view, including color-coded UAV paths, revealed targets, and an event manager with target events displayed on urgent and solved tabs. The right screen simultaneously presented live camera views from the four separate UAVs. The objective of the task was to work with the UAVs to identify and classify potential targets along each route as friendly, neutral, or enemy assets. Once completed, the participant would select the route with the least amount of enemies to send supplies through to their allies. Each route was in a desert environment with town-like features such as various types of houses, tents, and desert-like foliage. Based on previous research, three or more UAVs that require monitoring from the human operator can lead to performance decrements (Porat et al., 2016; Humann and Pollard, 2019). Therefore, the current study utilized a 4:1 UAV-to-operator ratio to allow for differences in operator performance with LOAs to emerge. Each UAV would identify and classify targets along their route per the experimental condition. Each of the four scenarios had 11 friendlies, 10 enemies, and 3 neutral targets. This ratio was selected to be in line with previous research, while still allowing for performance variations to emerge (Ruff et al., 2002; Chen and Barnes, 2012; Wohleber et al., 2019; Rebensky et al., 2021a). Targets were evenly distributed across each scenario and the UAV route free of enemies changed every scenario. The following additional events were layered in the study.

Reliability of the UAVs’ object detection was set to approximately 92%. The reliability of UAV’s object classification of the targets was also set to approximately 92%. This is similar to other UAV studies (e.g., 86%, Wohleber et al., 2019; 90%, Chen and Barnes, 2012; 95%, Ruff et al., 2002) and was chosen as automation will always be, to some extent, imperfect but needs to be higher than 70% to still be utilized by operators (Wickens and Dixon, 2007).

In the current study, the UAVs were responsible for collision avoidance and automatically flew. This allowed participants to take manual pictures if desired. Furthermore, each scenario consisted of two mislabeled targets and two missed targets. For example, if an agent found an enemy target, but labeled it as a friendly target, the target was mislabeled. If the UAV flew by an enemy target and did not take a picture of the target, it would be considered a missed target. The UAV agents that made missed target errors and mislabeled errors were changed per scenario so that performance of one particular UAV was not consistently worse comparatively. As a parallel task, participants were asked to select a route free of enemy assets to send a friendly convoy at the end of the mission. Two of the 26 targets were missed by the agents, equating to approximately 92% reliability for object detection. Two out of the 24 targets found by the agents were mislabeled, equating to 92% reliability for object classification. At the end of the mission, participants chose a route free of enemy assets to send a friendly convoy. The road free of enemy assets would change every scenario to provide counterbalancing measures.

### Experimental Setup

**FIGURE 2:**
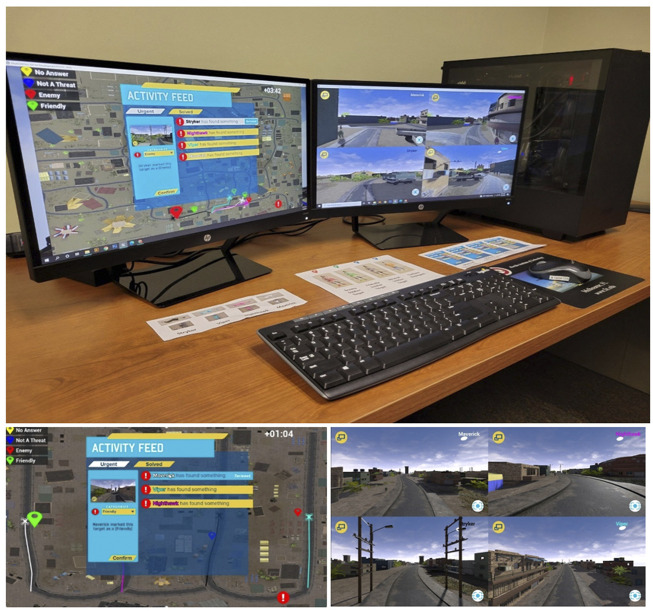
Experimental testbed.

The simulated task was completed on a custom-built desktop computer equipped with a Ryzen 7 3800X CPU, 32GB of RAM, a NVIDIA 2080ti graphics card, a Windows 10 Home operating system, two monitors positioned side by side, and a Logitech wireless keyboard and mouse (see Figure 2). The simulation multi-Unmanned Aircraft Vehicle Simulator for Teaming Research and Evaluation of Autonomous Missions (multi-UAV STREAM) was created in Unreal Engine 4 in conjunction with the Air Force Research Lab’s Gaming Research Integration for Learning Laboratory.

### Measures

Demographic information collected included age, gender, level of drone experience, and level of gaming experience. The following measures were collected for each of the research question areas.

#### Mission Performance and Operator State Measures

Mission performance has been classified in other UAV or multi-HAT studies as the following: 1) the number of targets correctly detected and identified (Chen and Barnes, 2012; Chen, 2010; Ramchurn et al., 2015; Rebensky et al., 2021a), 2) number of mistakes (Porat et al., 2016), 3) number of missed targets (Porat et al., 2016), and 4) mission map accuracy (Chen, 2010). In the current study, the number of correctly identified targets was utilized as it encompassed all three elements of correct targets, incorrect targets, and the accuracy of the types of targets marked on the mission map. Additionally, at the end of each mission, participants were asked to select a safe route to send the caravan. Performance on this task was used as an additional decision-making mission performance metric. To assess operator stress levels, the Short Stress State Questionnaire (SSSQ) distress subscale was utilized, which has been used in other UAV studies (Matthews et al., 2005; Dasgupta, 2010; Wohleber et al., 2019). To assess operator mental workload, the widely used NASA-Task Load Index (NASA-TLX) was administered (Hart, 2006; Richards, 2020).

#### Trust Measures

To measure subjective perceptions of trust in each LOA, a modified measure from Muir’s (1989) framework of trust was utilized, similar to studies by Master et al. (2000), Lee and Moray (1992), and Du et al. (2019). However, the following adjustments to Du et al.’s (2019) were made: 1) the word “autonomous vehicle” was replaced with “autonomous agents” and 2) the rating system of “none at all” to “extremely high” on a scale of one to seven was replaced with anchors of “not at all” and “completely” and a 10-point scale from the study by Lee and Moray (1992) to allow for more variability and the inclusion of complete trust in participant ratings. For behavioral indicators of trust, in driving simulation studies, taking over manual control in a potential collision or missed exit indicated a low level of trust in the automation to follow through (Miller et al., 2016). Therefore, the current study utilized the number of times an operator took manual pictures as an indicator of distrust in UAV teammates.

#### Team Effectiveness Measures

Perceived team effectiveness measures are important to target not only actual performance but human perceptions of performance as well. “Evaluations of HAT designs necessitate methods and metrics for assessing effectiveness and efficiency, yet such metrics are currently lacking” (Strybel et al., 2018, p. 25). Therefore, team effectiveness was measured using a self-developed singular question of “How effective do you believe your team was in completing the mission?” rated on a 5-point Likert scale from 1 = not at all effective to 5 = extremely effective. Additionally, at the end of the study, participants were asked again “How effective was the (manual/advice/consent/veto) condition in assisting you to complete your mission?” for each condition rated on a 5-point Likert scale from 1 = not at all effective to 5 = extremely effective. Open-ended responses were asked in tandem with end-of-study effectiveness questions. The open-ended questions asked the participant to describe what they liked or disliked about the condition, and why. Team coordination was assessed using a single question from the coordination subscale from the study by Mathieu et al. (2019): “to what extent does our team actively work to coordinate our activities with one another?” rated on a scale of 1 = “not at all” to 4 = “to a very great extent.”

### Procedure

Participants were recruited *via* email, flyers, and social media to partake in the study. Once participants arrived, they were asked if they experienced any COVID-19 symptoms within the past 14 days. After this, their body temperature was taken for precautionary measures, and they were informed that they were to wear a mask for the duration of the study. All equipment was sanitized between participants, and hand sanitizer was provided for their convenience. Participants were then asked to sign an informed consent form and given a pre-survey, where they were asked demographic questions, their levels of experience playing video games, level of drone experience, and given the propensity to trust in technology scale.

After completing the pre-survey, participants entered the training segment of the study. In the training segment, a slideshow was presented in which the different types of autonomy levels (manual, advice, consent, and veto) were explained and illustrated using both video and text. Videos illustrated the use of different features such as photo enlargement, manual photographing, and utilization of the event manager. Participants were advised to take their time reviewing the slideshow and encouraged to watch the videos provided. The training segment was self-paced; however, participants typically spent approximately 10 min on this section. After participants completed the training slideshow, the participants were given a 3-min-long training mission, in the manual condition. This included one neutral, friendly, and enemy asset per drone route, totaling up to 12 targets in the training scenario. Participants classified, categorized, and confirmed every target whilst monitoring four drones simultaneously. This abbreviated scenario allowed participants to familiarize themselves with the controls and experimental tasks to prepare them for the upcoming missions.

Participants were then notified that the missions would last 5 min, followed by questionnaires after each mission. Furthermore, participants were informed that their mission goal was to scope out enemies and find a clear route to send a caravan. Finally, participants were told that the UAVs would have a target detection and classification reliability of approximately 90%. Then, participants completed the four experimental missions in their respective counterbalanced order. After each scenario, participants were asked to fill out an online questionnaire including the SSSQ Distress Subscale, NASA-TLX, trust questionnaire, individual agent trust ratings, communication effectiveness, team effectiveness, and open-ended comments. After the last post-task survey was completed, the participants were asked to complete a questionnaire capturing their perceived effectiveness for each LOA and open-ended comments. Data were then exported into SPSS for data analysis.

## Results

The following section presents the results of analyses related to 1) operator performance, stress, and workload, 2) trust, and 3) perceived effectiveness. All variables included in analyses were checked for normality *via* Q-Q (quantile-quantile) plots. In addition, a correlation matrix was checked to ensure linear relationships between the dependent variables included in analyses together while also ensuring multicollinearity was not occurring.

### Mission Performance and Operator State

#### Performance, Stress, and Workload

To determine how well participants performed in each LOA and the resulting impacts on stress and workload, the following measures were utilized in a repeated measures MANOVA: 1) the total percentage of correctly identified targets, 2) stress as measured by the SSSQ distress subscale, and 3) workload as measured by the NASA-TLX. The null hypothesis was as follows: LOA will have no effect on performance, stress, and workload. The analysis revealed a significant overall MANOVA model, *F* (9, 29) = 3.71, *p* = 0.003. Univariate follow-up analyses revealed significant influences on performance *F* (2.41, 89.15) = 5.59, *p* = 0.003, η^2^ = 0.13, workload *F* (3, 111) = 5.08, *p* = 0.002, η^2^ = 0.12, and stress *F* (3, 111) = 9.79, *p* < 0.000, η^2^ = 0.21 by LOA.

*Post hoc* analyses for performance revealed that participants performed significantly worse in the manual condition than in the consent (*p* = 0.001) and veto (*p* = 0.015) conditions. The same was true when comparing the advice condition to the consent (*p* = 0.016) and veto (*p* = 0.017) conditions. However, there were no significant differences between manual and advice conditions or between consent and veto conditions relative to performance. The results indicate significantly better performance in higher levels of autonomy than in lower levels of autonomy.

Similar trends were observed for stress levels with significantly higher stress levels observed in the manual condition than in the consent condition (*p* = 0.003) and veto condition (*p* = 0.015). There were also significantly higher stress levels in the advice condition than in the consent (*p* = 0.010) and veto (*p* = 0.014) conditions. No significant differences in stress levels were found between the manual and advice conditions or between the consent and veto conditions.

With respect to workload, the manual condition resulted in significantly higher workload scores than the consent (*p* = 0.001) and veto (*p* = 0.000) conditions. The advice condition also resulted in significantly higher workload than the consent (*p* = 0.002) and veto conditions (*p* = 0.001). No significant differences in workload were observed between manual and advice or between consent and veto (see Table 3).

**TABLE 3:** Mission performance scores by condition.

Construct	Captured by	Average scores by condition			
		Manual	Advice	Consent	Veto
Performance	*Percentage of correctly detected targets*	84.27%	85.00%	89.10%	88.54%
Stress	*SSSQ Distress subscale score*	10.12	10.58	7.43	8.04
Workload	*NASA-TLX Score*	61.17	60.78	54.04	52.56

#### Additional Performance Metrics

**FIGURE 3:**
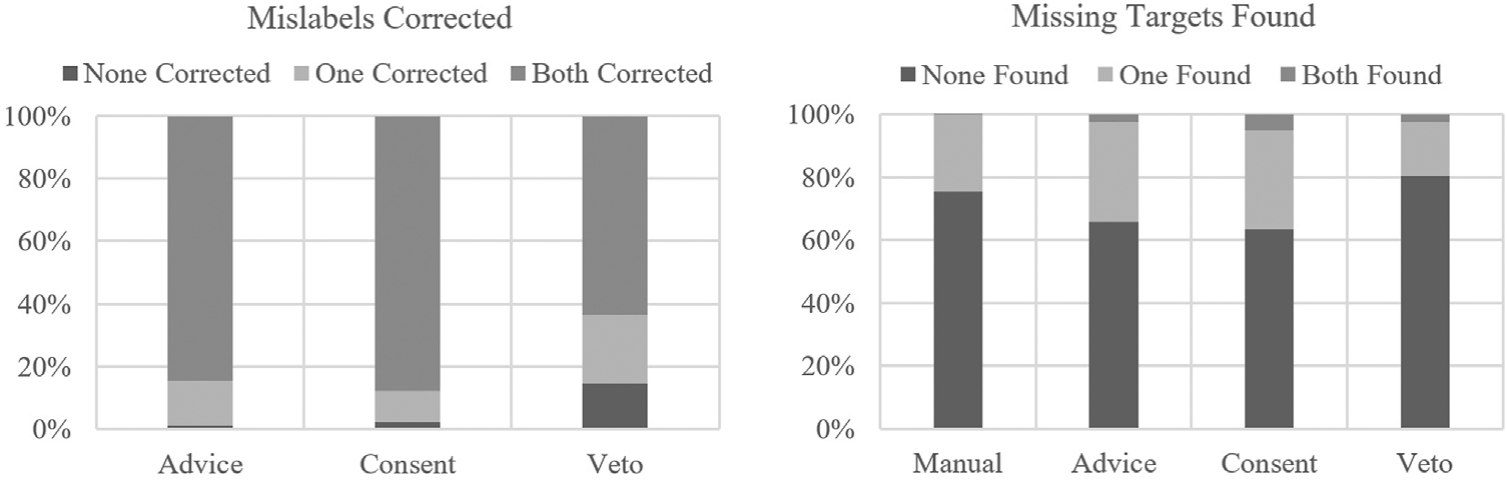
Mislabels corrected and missing targets found by condition.

Participants were informed that the agent was not 100% reliable and the agents would make mistakes in both target detection and classification approximately 1 in 10 times. Each condition had two mislabeling events and two missed target events. Mislabels, or agent mistakes relative to target classification, were not present in the manual condition as the agents only assisted with target detection in the manual condition. As LOA increased, more participants failed to correct mislabels, with the highest percentage of mislabels going uncorrected in the veto condition. For missing targets, more missing targets were found in advice and consent than in the manual. In the veto condition, there was a decrease in the percentage of missing targets found compared to the advice and consent conditions, and even less than the manual condition (see Figure 3).

An additional measure of performance included whether or not participants selected the correct route. This was analyzed separately as it was nominal in nature of whether the participants selected the correct route or not. In the manual condition, the incorrect route was selected four times or 9.8% of the time, followed by zero times or 0% of the time in advice, one time or 2.4% of the time in consent, and two times or 4.9% of the time in veto.

### Trust

**FIGURE 4:**
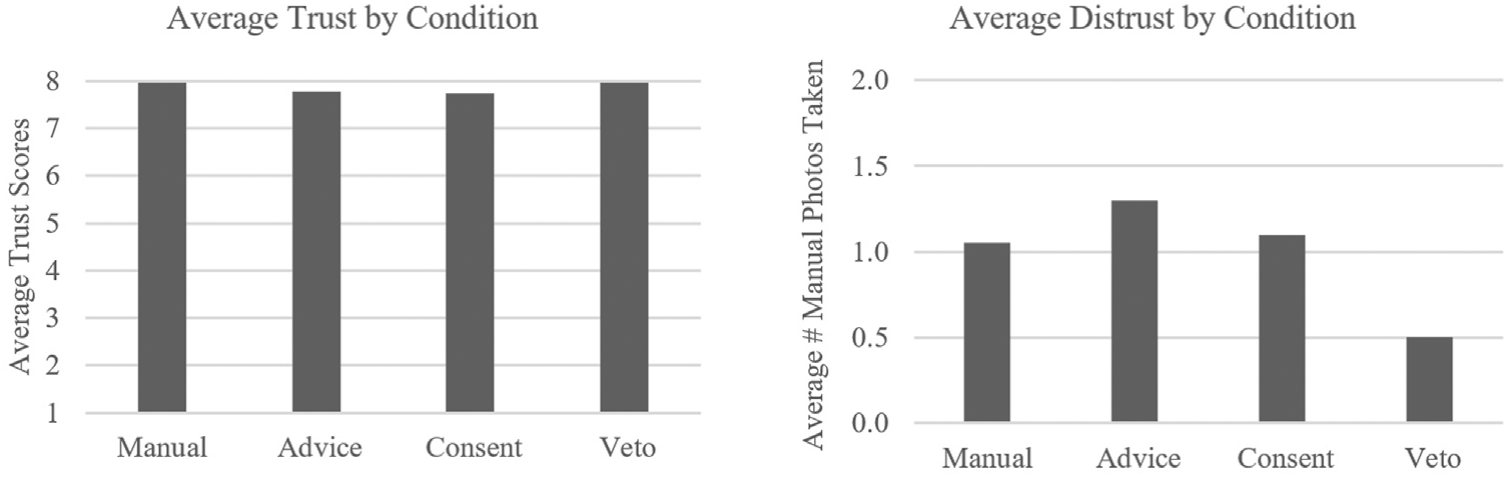
Average trust and distrust ratings by condition.

To determine the impact of each LOA on participant perceived trust in their agent teammates, the following measures were utilized in a repeated measures MANOVA: 1) trust as measured by the modified Du et al.’s (2019) measure of trust and 2) manual pictures as a measure of distrust. The null hypothesis was as follows: LOA will have no effect on trust. The analysis revealed a non-significant MANOVA model, *F* (6, 35) = 2.24, *p* = 0.06. However, if the reader is willing to accept a *p* value of 0.06, there was a noticeable effect of LOA on distrust. Univariate follow-up analyses revealed no significant influences of LOA on trust *F* (3, 120) = 1.34, *p* = 0.262, η^2^ = 0.033, but a significant influence of LOA on distrust, *F* (3, 120) = 2.68, *p* = 0.039, η^2^ = 0.067. Inversely, the highest levels of distrust, by means of manual photo taking, were reported by participants in the advice and consent conditions, with highest levels of distrust found in advice (see Figure 4).

### Team Effectiveness

**FIGURE 5:**
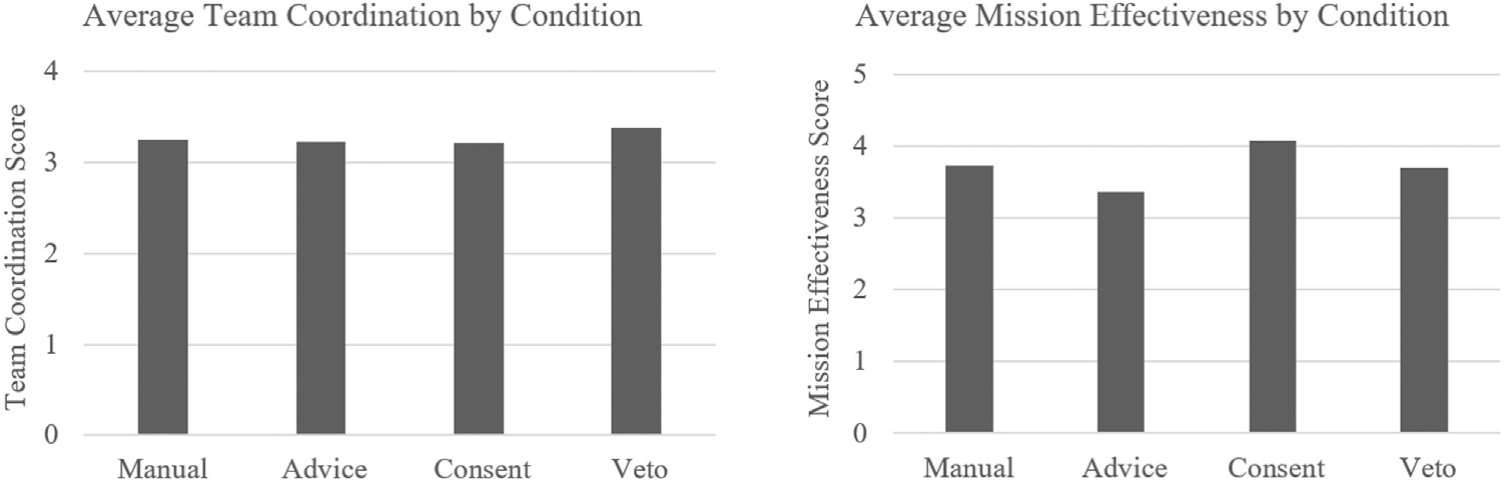
Average team coordination and mission effectiveness scores by condition.

Perceived effectiveness of the team was captured using the end-of-study subjective measures of team effectiveness and post-task measure of team coordination. The null hypothesis was as follows: LOA will have no effect on team effectiveness. Team effectiveness for the advice condition was rated “somewhat effective” with an average score of 3.3, followed by “somewhat” to “very effective” for the manual and veto conditions with an average score of 3.7, and ratings of “very effective” for the consent condition with an average score of 4.1. An ANOVA revealed significant effects of LOA on team effectiveness ratings *F* (2.10, 8.12) = 3.45, *p* = 0.034, η2 = 0.08. *Post hoc* analyses revealed that the advice condition was rated as significantly less effective than the manual (*p* = 0.03) and the consent condition (*p* < 0.001). No significant differences were found between manual and consent, manual and veto, advice and veto, or consent and veto. For team coordination, ratings had a very narrow range across conditions from 3.21 to 3.38, indicating an ability to coordinate with their agent teammates “to some extent” (see Figure 5).

## Discussion

### Mission Performance and Operator State Findings

For performance, stress, and workload levels, the results indicate significantly better scores in the two higher levels of autonomy than in the two lower levels of autonomy. In line with previous research, the higher levels of automation resulted in lower levels of stress and workload (Chen and Barnes, 2014; Barnes et al., 2015). A small increase in stress in the veto condition compared to the consent condition could be due to the change in the nature of the task from an active target identification task to a supervisory role. Some participants noted “I liked this mode the least. I did not like not having any control and just reviewing” and “I did not like the drone making the choices for me. I [would] rather confirm first before the drone does.” The nature of placing more tasking on the automation, but the human being ultimately responsible for its actions, may have elicited a small increase in stress. Additionally, advice may not have resulted in any reductions in stress or workload as the identification task was still primarily the responsibility of the human. One participant noted that “advice mode was effective but I still had to perform the task of categorizing each target which became time consuming making me pay more attention to the picture.” Another noted “I still have to manually check the picture so it still feels like manual, although I have an idea on what to look for.” These comments are in line with certain HHTs research, which found that when a team member shares the workload, this can lead to less stress and better performance (Chen and Barnes, 2014).

Similar to Ruff et al. (2002) and Barnes et al. (2015), we found that performance, across the different metrics for performance, was best for the middle levels of autonomy. Higher levels of autonomy led to better target identification accuracy, reducing workload, and reducing stress; however, consent was superior when compared to veto for correcting agent mistakes and decision-making. The consent condition allowed for the higher percentage of mislabels and missing targets detected. The added assistance of pre-marking the target may have allowed for additional workload reductions that allowed the participant to play a more active role in supervising the agents. In the veto condition, it is possible that the participants became more complacent with the agents and did not catch as many mistakes as they could have. As a result, in the consent condition, participants had the most favorable performance related to correct targets, missing targets found, mislabeled targets corrected, and correct routes identified.

Interestingly, the incorrect route frequency findings do not align with the target accuracy percentages observed in the current study. Based on the percentage of correct targets, it was anticipated that veto and consent would lead to the highest decision making accuracy as their mission maps of enemy and friendly target locations were the most accurate. However, it was advice and consent that led to the lowest frequencies of incorrect routes selected. The findings suggest the middle levels of autonomy, advice and consent, sufficiently support operator workload while also keeping the human sufficiently in the loop to make accurate battlefield decisions. Similar to findings in previous research, higher levels of autonomy can lead to automation bias, or an overtrusting of agent behaviors resulting in poorer performance in terms of mislabels corrected and missing targets found (Barnes et al., 2015; Chen et al., 2016). As a result, it is suggested that the lower-level tasks such as object detection fall on the automated agents, whereas tasks such as object identification require human-in-the-loop consent and the human to make the ultimate decision to avoid automation complacency—particularly in military settings (Arciszewski et al., 2009; Barnes et al., 2015). The consent condition allowed for reduced workload, giving it clear gains over the manual and advice conditions, without human-in-the-loop challenges that occur when automation becomes too high in the veto condition.

For performance, future research should examine offering confidence ratings of agent object identifications to help operators determine which events require reviewing and which do not. This may help with complacency issues in veto designs. Additionally, methods to improve the advice condition are still needed. In the current study, advice did not lead to significant improvements in workload, stress, and performance from the manual condition—even though the advice condition assisted with a whole additional task of target classification. Participants enjoyed the extra step that consent took by premarking the targets. It is possible that based on the type of task and the riskiness involved, advice may be ideal in some tasks whereas consent may be ideal in others (i.e., consent for ISR missions, but advice for missile strike missions; or swapping between the two during periods of high or low workload; Arciszewski et al., 2009). As a result, future research should look at methods for improving the advice condition to bring its mission performance score closer to those observed for consent in the current study.

### Trust Findings

Relative to trust, the current study’s findings are consistent with previous research, finding no significant differences between various levels of autonomy. Du et al. (2019) reported similar results, finding no evidence that autonomous vehicles, which provided further explanations, led to higher trust ratings. Prior research has also noted that trust can change regardless of team performance, so trust in different LOA conditions may be a construct that is not impacted by the team structure (Mcneese et al., 2021). Aside from Du et al. (2019)’s findings, impacts of LOA on trust in an agent have been scarcely explored, a gap that this effort aimed to address (Beer et al., 2014; Mcneese et al., 2021). As agents become more prevalent, particularly starting with advice as the most likely LOA, it is important to note the impacts of these LOA designs. Participants reported that in the manual condition, they felt as though they were more “mentally involved with this mode” and “trusted (themselves) to correctly identify soldiers.” In this condition, the human is given all responsibility related to object identification; hence, the participants also felt as though they were more “involved in the decision making process,” indicating a stronger sense of control and trust with the agent team. In the advice condition, participants reported that there was “slightly less workload” than in the manual condition, but it was “not as useful” because although the agent was assisting with target identification, the participants “still had to perform the task of categorizing each target which became time consuming.” As a result, participants reported that they would just “have to trust the drone” if they did not have the time to double check its advice, indicating a lack of control in the identification process. Comparable reports were found in the consent condition, with participants indicating they “had a similar issue with this condition that (they) had with the advice condition” in the sense that they “still felt the need to review all of the pictures.” However, the change in trust observed in the consent condition could be due to the fact that participants had “a more relaxed pace” to make the decisions. A few participants reported that the condition “reduces trust in the system” by “having to go back and review the decisions of the agents,” indicating a lack of control and an absence in the decision making process. A majority of participants reported that the veto condition aided their ability to “scan and monitor” the UAVs, allowing for more time to catch any incorrect target identifications. They also reported a “decrease in workload” due to an even more relaxed pace. The increased time to scan and monitor the UAV paths led to the marginally increased trust reporting over advice and consent. However, it should be noted that mislabels and missed targets were detected less often in the veto condition, contrary to participant comments. This disruption in trust could be due to an interaction between workload and perceived responsibility by the human teammate. With the decreased workload and increased ability to scan for targets, participants also have more time to take manual pictures. In the manual condition, the level of workload impacted the participants’ ability to take pictures due to workload levels. The advice and consent condition offered more time for the participants to scan and subsequently take pictures. One would anticipate even more manual pictures in the veto condition based on this trend. However, in the veto condition, the automation is responsible for both target detection and identification; thus, the human is less involved. It is possible, as a result, that participants became over-trusting and more complacent as indicated by the performance of missed targets detected, mislabels corrected, and incorrect routes selected in the veto condition—which was poorer than the advice and consent conditions.

Although both advice and consent conditions require the participant and agent to work in tandem to correctly identify targets, the diction used in each condition differed. In the advice condition, the agent reported what it *thinks* the target should be labeled (friendly, enemy, or not a target). In the consent condition, the agent reported that it *marked* a target (as either friendly, enemy, or not a target). This difference in diction could explain the highest level of distrust being for the advice condition. The advice condition may spark the thought of *why?* with the advice condition appearing less transparent, whereas participants may be more willing to accept the consent condition at face value. The explainability of an autonomous agent, also known as explainable AI, is a form of autonomy in which the human explicitly understands and can easily make sense of the autonomous agent’s workings (Shin, 2021). It is reliant on the autonomous agent’s ability to inform the human *why* it is doing something, which is a notion that is extremely obvious and prominent in HHT, but difficult to employ in HATs (Shin, 2021). Therefore, this concept of explainable AI requires the automation to be transparent in its decision-making process (Endsley, 2015). Transparency leads to a shared mental model and awareness between the human and agent, allowing for a more predictable, dependable, and efficient team dynamic (Endsley, 2015; Schaefer et al., 2019). In all conditions, the automation never explains *why* it chose a certain target, thus leaving the human out of the loop in its identification processes. The absence of this transparency within these conditions may explain similar levels of trust between conditions, as indicated by both subjective trust ratings as well as by distrust measures. This may be particularly true for the advice condition in which the agent teammates stated what it *thinks* the target is. This condition may have inadvertently triggered a doubt or questioning in participants of *why* it thinks it is a particular target. As participants had to select the target type itself, many participants noted second guessing themselves when an agent made an incorrect suggestion. However, the same participants noted the consent condition was quicker and more accurate even though all conditions exhibited the same level of accuracy. As participants were forced to actively choose to select a target type against the agent’s suggestions as opposed to changing or confirming the agent’s automatic decision-making in consent and veto, there may have been more ruminating on the agent’s reliability in the advice condition. In the veto condition, where the automation is completing the identification task in full, we see a marginal increase in trust. In cases where the LOA is high, participants may tend to over-trust the automation, leading to over-reliance in situations where its capabilities are not adequate (Lee and See, 2004). The effects of too much automation have been observed previously and is a notion that does not commonly occur when dealing with solely HHTs (Biros et al., 2004). However, due to the lack of significant differences in trust measures found in this study, there is the possibility that trust is not directly impacted by LOA in this particular setting.

For trust, future research should focus on developing explainable advanced intelligence (AI) within each or some of the conditions to gain a better understanding of how the level of transparency impacts a human’s trust in different LOAs. Future work should also take into consideration the diction used within each condition, as the wording of the agent’s decision making can impact a person’s understanding or perceptions of how the agent identified targets. These design improvements could lead to a method that allows the agent to decide when to employ explanations to the human teammate and the correct way to do so, which could lead to an increase in overall trust. Considering advice is the most logical starting point as agents make the pivot from tool to teammate, it is clear that many improvements are needed to make advice a viable option for warfighter agents. Furthermore, a more in-depth qualitative analysis focusing solely on the dimensions of trust (competency, responsibility, reliability, faith, predictability, and dependability) in different LOAs could lead to a better understanding of how to design an agent that is trustworthy in a HAT domain.

### Team Effectiveness Findings

In the current study, it was found that the consent condition offered the sweet spot of human involvement and performance gains. This was reflected in consent receiving the highest effectiveness ratings. One participant noted “Consent was the most effective. The dropdown choice was already selected which saved a lot of time and attention.” Meanwhile, for the other conditions, the participant noted “the manual required a lot more time and attention,” “the advice was not any more effective than manual,” and “the veto condition felt rushed...need to go back and confirm.” Ultimately, the participant stated they “like consent where the human participant is still involved in the decision before it is made.” Other participants noted that consent was “the perfect balance of teamwork between the operator and the agent.” However, variations in perceived team coordination were not demonstrated in the current study. Very little research has been done related to the perception of camaraderie and the sense of “team” in HAT. It is possible that the interaction method utilized in the current study still felt more “tool-like” than “teammate-like” to participants. Additionally, this measure had between 10 and 20% missing data per condition. This was not the case for any other question in the study and could indicate some inability to rate their perceptions of team coordination potentially due to it not being a construct they felt was occurring in the team structure. Additionally, it could be possible that the single item measure was not diagnostic of team coordination.

For team effectiveness, future research is needed to explore the impacts of different interaction methods (adaptive and adjustable automation), conversational methods (the way agent decision making assistance is worded), and interactions (the way the human supervises and interacts with agents), particularly, the effect of each of these designs over time and the subsequent changes in the perception of the team in HATs. These methods may allow for more variation in perceptions of team coordination than the method utilized in the current study. The current measure also consisted of only one measure that exhibited a high level of skipped responses. It is possible that measures of HHT coordination are not sufficient as measures of HAT coordination and more effective measures of HAT team perceptions are needed.

## Conclusion

The evolution of multi-HAT operations has unveiled areas in need of improvement in agent design. This study addressed current research questions in regards to HAT performance as agents traverse the LOA continuum and the associated challenges humans experience. The impact of LOA in UAV teammates on team effectiveness, team coordination, trust, decision making, stress, and workload revealed the need for trustworthy system design strategies to improve multi-UAV teams for future HAT operations. Participant’s performance, stress, and workload scores indicated that the two higher levels of autonomy resulted in lower levels of stress and workload, and thus overall better performance. However, decision making and detection of agent mistakes in the veto condition indicated issues with automation complacency and out-of-the-loop challenges. The same can be said for team effectiveness, with the second-highest LOA possessing the highest-rated effectiveness scores, potentially due to the human and agent responsibilities being balanced to alleviate workload while still keeping the human informed. Although no significant differences were found in trust scores between the LOAs, the study identified key areas of trust characteristics that require further investigation in order to establish a trustworthy multi-UAV HAT. As the intelligence of automated teammates increases, redesigning agents to better support humans will aid in improving HAT in the multi-HAT domain.

## Data Availability

The raw data supporting the conclusions of this article will be made available by the authors, without undue reservation.
